# Associations between loneliness and frailty among older adults: Evidence from the China Health and Retirement Longitudinal Study

**DOI:** 10.1186/s12877-022-03044-0

**Published:** 2022-07-01

**Authors:** Sha Sha, Yao Pan, Yuebin Xu, Lin Chen

**Affiliations:** 1 School of Sociology and Population Studies, Nanjing University of Posts and Telecommunications, Nanjing, China; 2 School of Social Development and Public Policy, Beijing Normal University, Beijing, China; 3 Institute of Advanced Studies in Humanities and Social Sciences, Beijing Normal University at Zhuhai, Zhuhai, China; 4 Beijing Normal University at Zhuhai, Zhuhai, China

**Keywords:** Frailty, Loneliness, Cross-lagged analysis, Older adults

## Abstract

**Background:**

Previous studies have demonstrated the associations between loneliness and frailty in late life. However, there is a lack of consensus on the direction of the relationship. The present study aimed to examine the interdependencies between loneliness and frailty over time.

**Methods:**

Data on participants aged 60 years old and above were collected from the 2011, 2013, and 2015 samples of the China Health and Retirement Longitudinal Study (CHARLS). Loneliness was measured by a single question from the Centre for Epidemiological Studies Depression scale, and frailty was assessed by the Physical Frailty Phenotype (PFP) scale. Cross-lagged panel models were utilized to examine the potential bidirectional relationship between loneliness and frailty.

**Results:**

Reciprocal associations were found between loneliness and frailty. Furthermore, we found that baseline frailty and early change in frailty had a significant predictive effect on late change in loneliness. Higher baseline loneliness in older adults may create a potentially vicious cycle that influenced early change in frailty and continued to cause late change in loneliness.

**Conclusion:**

A bidirectional relationship may exist between loneliness and frailty among older Chinese adults over 60 years old. Lonely older adults should be alerted to the potential self-reinforcing cycle of loneliness that affects their health.

**Supplementary Information:**

The online version contains supplementary material available at 10.1186/s12877-022-03044-0.

## Introduction

China has the largest population in the world and has also been aging at a significantly faster rate than other low- and middle-income countries [1]. According to the National Bureau of Statistics of China, the country has nearly 264 million people over the age of 60, accounting for 18.7% of the total population by the end of 2020. The challenges of an aging society stem not entirely from the number or proportion of the older population but also from the health status of the elderly.

Frailty and loneliness are important indicators of the health status of older adults. Frailty is characterized by the impairment of multiple related physiological systems and heightened vulnerability to different stresses [2, 3]. Frailty is also a more effective indicator of health outcome risks than chronological age [4] and has been proven to lead to a variety of adverse health outcomes, such as disability [5], hospitalization [6], institutional care [7], and mortality [8]. Loneliness, which is often defined as subjective and unpleasant feelings that arise from the perceived deficit between the quantity and quality of one's preferred and actual relationships, is becoming a public health concern [9–11]. Previous studies have shown that loneliness is a major risk factor for health outcomes, and may accelerate physical aging [12], impair cognitive ability [13], affect physical function status [14], and increase the risk of diminished longevity in old age [15]. Both frailty and loneliness warrant more attention in health and social care systems.

Many longitudinal studies have shown that loneliness influenced the onset of frailty in old age and the transition of frailty in older adults: (1) one study used the Physical Frailty Phenotype (PFP) scale to measure frailty and the UCLA loneliness scale to measure loneliness. They found that higher loneliness was significantly associated with an increased risk of becoming frail or prefrail in follow-up [16]; (2) two other longitudinal studies assessed frailty in the PFP scale and the FRAIL scale, and measured loneliness using the Revised UCLA Loneliness Scale and one-single question, respectively. They found that a high level of loneliness was positively related to the worsening of frailty status over time and negatively related to the recovery of prefrail or frail older adults [17, 18]. Activity engagement may play a role in explaining the effect of loneliness on frailty. Lonely people were more likely to be inactive, which increases the risk of frailty in older adults [19, 20]. The inflammatory system is a channel through which biological processes mediate this relationship. Specifically, loneliness affects the inflammatory system [21], which provides a physiological basis for the geriatric syndrome of frailty [22].

There is also evidence of the effect of frailty on loneliness in older adults. Cross-sectional studies that both used the PFP scale to measure frailty and the De Jong-Gierveld Loneliness Scale to measure loneliness had shown that prefrail and frail older adults had a higher level of loneliness than their nonfrail peers [23–25]. Another cross-sectional study using the UCLA Loneliness scale also showed a progressively high prevalence of loneliness with increasing frailty [26]. A longitudinal study that used the PFP scale and the De Jong-Gierveld Loneliness Scale found that baseline frailty status was related to an increase in loneliness at follow-up [27]. The underlying mechanism through which frailty may affect loneliness is unclear. However, previous studies have suggested that poor physical health may lead to fatigue and mobility difficulties, which are harmful to the establishment and maintenance of satisfactory social networks and thus contribute to loneliness [28, 29].

While evidence is accumulating for the existence of the relationship between loneliness and frailty, in subject to research design (e.g., cross-sectional design) or construction, most studies have focused on only one possible direction in the relationship, which has led to inconsistencies regarding the causal relationship between loneliness and frailty. Furthermore, previous findings are mainly based on population in Western countries, and little is known about the relationship in developing countries in Asia. This study aimed to provide additional evidence on the potential bidirectional association between loneliness and frailty using a cross-lagged panel design. Moreover, using data from a nationally representative sample of China [30] may contribute to the current understanding on associations between loneliness and frailty.

In the present study, we examined the association between loneliness and frailty in Chinese older adults (≥ 60 years old). Based on the research gap identified above, our first hypothesis is that there is a reciprocal relationship between loneliness and frailty in older adults (Hypothesis 1). In addition, the effect of loneliness on frailty transition has been proven [17, 18], but fewer studies have focused on the effect of frailty on changes in loneliness. Additional research is needed to examine the interaction between the changes in loneliness and frailty because if such an association does exist, then the change in one health domain can be an earlier warning the other, which is important for early health interventions in older adults. Therefore, our second hypothesis is that there is an association between changes in loneliness and changes in frailty in older adults. We investigated the relationship between loneliness and frailty in older adults from a dynamic change perspective: 1) changes in loneliness would be related to changes in frailty (Hypothesis 2a); 2) changes in frailty would be related to changes in loneliness (Hypothesis 2b).

## Methods

### Study Participants

Data were obtained from the China Health and Retirement Longitudinal Study (CHARLS), which aimed to collect high-quality nationally representative data of Chinese households and individuals aged 45 and above to analyze the aging of China [30]. Samples were selected by a four-stage, stratified, cluster sampling design, covering 150 county-level units and 450 village-level units [31]. The CHARLS was approved by the ethical review committee of Peking University and all participants gave written informed consent. Details on the CHARLS survey design can be found in previous study [32]. All methods were performed in accordance with the relevant guidelines and regulations.

The baseline survey, in which 17,596 community-dwelling residents participated, was conducted in 2011 (wave 1). It was followed by the 2013 survey (wave 2, 18,455 participants), 2015 survey (wave 3, 20,967 participants), and 2018 survey (wave 4). In this study, we used wave 1, wave 2, and wave 3 since wave 4 did not release enough information on frailty in older adults. Participants were included in the present study if they met the following criteria: (1) the participants were aged 60 years old or older at baseline and attended all three waves of interviews (*N* = 5,761); and (2) the participants provided enough information on their loneliness and frailty (*N* = 2,412). The baseline age range of the analytic sample of older adults was 60–93 years, with a mean age of 66.7 years. The majority of participants were male (53.2%,* n* = 1283) and from rural areas (68.5%, *n* = 1,653). Other sociodemographic characteristics are described in Additional Table 2.

### Measures

#### Frailty

Frailty was assessed by the Physical Frailty Phenotype (PFP) scale, which was developed from the Cardiovascular Health Study (CHS), and consisted of five criteria: 1) weakness, 2) slowness, 3) exhaustion, 4) low activity, and 5) shrinking[2]. Since the survey design was not identical, as was the design of the CHARLS survey in which only half of the randomized participants took part in the physical activity module, this assessment was developed and validated in the CHARLS to investigate frailty in older Chinese adults [5, 33–35]. Participant scores 1 point for each criterion is met, and the total score ranged from 0–5. Details on each of the five criteria are provided in Additional Table 1.

(1) Weakness was measured by grip strength using a hand-held mechanical dynamometer twice for each hand in a standing position. Participants met the weakness criteria if their grip strength was at or below the 20th percentile adjusted by gender and body mass index (BMI);

(2) Slowness was measured by the average time taken to walk a 2.5 m route two times. Walking speed at or below the 20th percentile adjusted for gender and height was viewed as slowness;

(3) Exhaustion was measured by two items of the Centre for Epidemiological Studies Depression Scale (CESD). Participants met the exhaustion criteria if they answered “Occasionally or a moderate amount of the time (3–4 days)” or “Most or all of the time (5–7 days)” to either of the two questions: “I felt everything I did was an effort” and “I could not get going”;

(4) Low activity was identified in participants if they answered “no” to three questions: “During a usual week, did you do any vigorous activities for at least ten minutes continuously”, “did you do any moderate physical effort for at least ten minutes continuously”, and “did you do any walking for at least ten minutes continuously”;

(5) Shrinking was identified in participants who self-reported to have lost five or more kilograms in the last year in wave 1 and wave 2. Participants in wave 3 met the shrinking criteria if their weight declined five or more kilograms between wave 2 and wave 3.

#### Loneliness

Loneliness was measured by a single question from the Centre for Epidemiological Studies Depression scale: “I felt lonely”. Single-question measurement of loneliness has been widely used. This type of measurement provided more direct access to participants' feelings of loneliness and has been proven to be valid and appropriate for the aging population [36]. The four-point response scale ranged from “1 = Rarely or none of the time (< 1 day)” to “4 = Most or all of the time (5–7 days)”. A higher score indicates a higher level of loneliness.

#### Covariates

We adjusted for covariates associated with frailty and loneliness, including age, gender (female = 0, male = 1), residence (rural = 0, urban = 1), education level (illiterate = 1, no formal education (received traditional Chinese school (i.e., Sishu) or did not finish elementary school but can read, write, etc.) = 2, elementary school = 3, middle school or above = 4), marital status (without spouse (widowed) = 0, with spouse (married or others) = 1), frequency of contact with children (seldom contact = 1, monthly contact = 2, weekly contact = 3), income (yuan/year), self-rated health (good = 1, so so = 2, bad = 3), number of chronic diseases, pain (0 = no, 1 = yes), smoking (0 = no, 1 = yes).

We also adjusted for activity participation frequency and cognitive ability. The CHARLS asked participants whether they had participated in the following activities in the past month and the frequency of their participation: (1) socializing with friends; (2) playing mah-jong, chess, cards, and going to community clubs; (3) providing free assistance to family, friends, or neighbors who do not live with you; (4) going to parks or other places to dance, to play sports, etc.; (5) participating in community-related organizations; (6) doing volunteer or charitable work; (7) taking care of a sick or disabled person who does not live with you; (8) attending educational or training courses; (9) investing in stock; and (10) using the internet. The frequency of participation in each activity was scored from 1 (not often) to 3 (almost every day), with 0 for non-participation. Higher total scores indicate more frequent participation. The CHARLS used episodic memory and mental intactness to assess cognitive ability. We added up the scores of episodic memory and mental intactness based on previous studies [37, 38], ranging from 0 to 31, with higher scores indicating greater cognitive ability (details on Additional Table 2).

### Statistical Analysis

The cross-lagged panel model is a common instrument to examine the potential reciprocal association between variables [39]. Thus, we implemented two cross-lagged panel models to test our hypotheses.

**Fig. 1:**
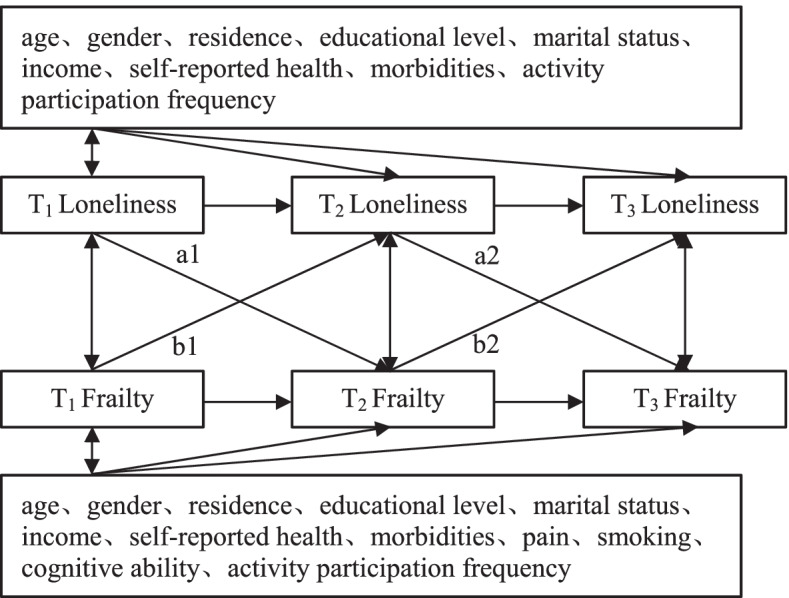
Conceptual diagram of cross-lagged associations between loneliness and frailty

We first employed a model to estimate the relationships between loneliness and frailty through repeated measurements of the same sample at three waves (T_1_, T_2_ and T_3_) (Fig. 1). Within the model system, we focused on the cross-lagged effect of loneliness on frailty (a1 and a2) and the cross-lagged effect of frailty on loneliness (b1 and b2).

**Fig. 2:**
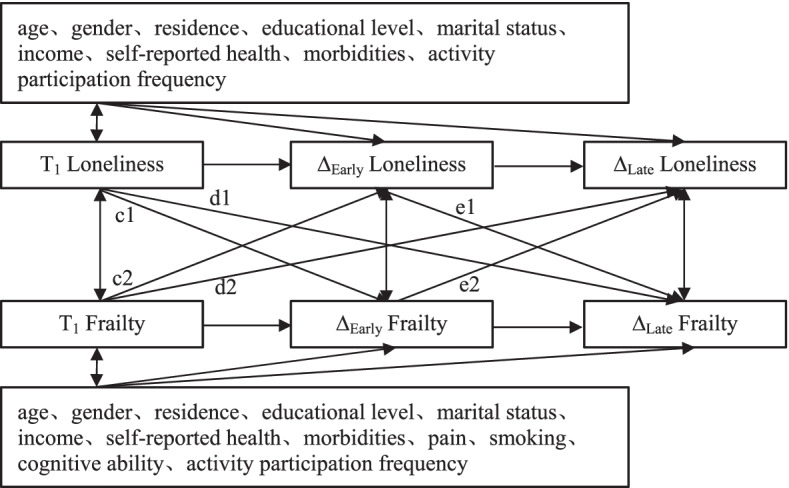
Conceptual diagram of cross-lagged associations between change in loneliness and frailty. ∆_Early_ Loneliness = T_2_ Loneliness–T_1_ Loneliness; ∆_Late_ Loneliness = T_3_ Loneliness–T_2_ Loneliness; ∆_Early_ frailty = T_2_ frailty–T_1_ frailty; ∆_late_ frailty = T_3 _frailty–T_2_ frailty

Then, we used a model to investigate the association between changes in loneliness and changes in frailty (Fig. 2). Based on the existing literature, we constructed early changes in loneliness and frailty as the difference between two measurements of the variable at Wave 2 (T_2_) and Wave 1 (T_1_) and late changes in loneliness and frailty as the difference between two measurements of the variable at Wave 3 (T_3_) and Wave 2 (T_2_) [40, 41]. Within the model system, we focused on: (1) the cross-lagged effect of T_1_ loneliness or frailty on early change and late change in the other variable (c1, c2; d1, d2); and (2) the cross-lagged effect of early change in loneliness or frailty on late change in the other variable (e1, e2).

We developed four possible cross-lagged panel models to examine the nature of the longitudinal relations between loneliness and frailty. First, the stability model (Model 0) included only the correlation paths and the autoregressive paths between loneliness and frailty at all time points. Second, we developed two unidirectional cross-lagged models, which were the cross-lagged path from loneliness to frailty in Model 1 (paths a1 and a2) and the cross-lagged path from frailty to loneliness in Model 2 (paths b1 and b2). By comparing the fitting results of Model 0 and Model 1, Model 0 and Model 2, we tested the cross-lagged association between loneliness and frailty. Third, a combined model (Model 3) was constructed based on the results of the comparison of the above-nested models (path a1, a2; b1, b2). Moreover, based on Model 3, Model 3–1 assumed that the cross-lagged path coefficients were equal across time (path a1 = a2; b1 = b2). The path of the relationship between loneliness and frailty was examined by comparing the results of Model 3 and Model 3–1. The factors influencing loneliness and frailty were not identical. When analyzing the relationship between loneliness and frailty, we included covariates that were not identified in the models with loneliness as the dependent variable and the models with frailty as the dependent variable (the models are schematically shown in Fig. 1 and Fig. 2).

In each nested model, we applied the full information maximum likelihood (FIML) method to accommodate all information from all observations [42]. Our model fit indices were the comparative fit index (CFI), root mean square error of approximation (RMSEA), and standardized root means square residual (SRMR). Models with values of CFI greater than 0.90 were considered acceptable; those with RMSEA and SRMA less than 0.08 had acceptable model fit, and those with RMSEA and SRMA less than 0.05 had good model fit [43, 44]. Model estimation was performed by robust maximum likelihood estimation (MLR), and the fits of the model were compared using the scaled χ2-difference test [45]. Stata 15.1 was used for basic statistical analysis, and Mplus 7.4 was used for model testing.

## Results

### Sample characteristics and correlations

The means, standard deviations, and pairwise correlations for loneliness and frailty across the waves are shown in Additional Table 3. Although most of the paired t tests in frailty and loneliness across three times were significant, the difference in mean values of the two variables across time was small. Additional assessment of the variations within participants over time in Additional Table 4 showed that 23.5% of older adults had increased frailty levels between T_1_ and T_2_, and 25.9% of older adults had increased frailty levels between T_1_ and T_3_. The loneliness of 14.4% of the older adults increased between T_1_ and T_2_, and that of 18.8% of the older adults increased between T_1_ and T_3_. Correlations showed a positive association between loneliness and frailty at each point of time; for example, a higher level of loneliness at T_1_ was associated with worsened frailty at T_2_ and T_3_, and vice versa.

### Model test of the relations between loneliness and frailty

The model fit statistics of the four models of the cross-lagged analysis can be found in Table 1. Based on the fit indices and the scaled χ2- difference test, we found that the model fit significantly increased after including the cross-lagged paths separately (Model 1, Model 2 compared to Model 0). This result suggested that the bidirectional relationship between loneliness and frailty was supported, thus we included with the two cross-lagged paths in Model 3. Subsequently, there was no significant decrease in the fit of Model 3–1 compared to Model 3. Thus, Model 3–1 provided the best and most parsimonious representation of the data (CFI = 0.930, RMSEA = 0.060, SRMR = 0.016).

**Table 1:** Model fit statistics of each of the four models of the cross-lagged analysis

Model	χ2	df	CFI	RMSEA	SRMR	ref	Δχ2 (Δ*df*)	*P*-value
0	160.343	18	0.917	0.057	0.019			
1	133.173	14	0.930	0.059	0.015	0	26.86(4)	< 0.0001
2	135.935	14	0.929	0.060	0.017	0	23.64(4)	< 0.0001
3	131.482	12	0.930	0.064	0.016			
3–1	133.878	14	0.930	0.060	0.016	3	2.44(2)	0.296

*Note,* Model 0 resembles the stability model with the correlation paths and the autoregressive paths between loneliness and frailty at all time points. In model 1 and model 2, we separately included the cross-lagged path from loneliness to frailty and the cross-lagged path from frailty to loneliness. In model 3, we included the bidirectional cross-lagged path based on the comparing results of model 0 and model 1, model 2. In model 3–1, we constrained the cross-lagged path coefficients in model 3 to be equal across time. χ2 is the model fit test statistic, df = degrees of freedom, ref. = reference model to compare with, CFI = comparative-fit-index, RMSEA = root mean square error of approximation, SRMR = standardized root mean square residual, Δχ2 (Δdf) is based on the scaled χ2-difference test

Table 2 shows the results of the standardized estimates of the relationship between loneliness and frailty (full model results are shown in Additional Table 5). After adjusting for covariates, the autoregressive paths of frailty and loneliness were stable across the three points in time, and the relationship strengthened as the path standardized coefficient increased over time (loneliness: T_1_ → T_2_: β = 0.25, *P* < 0.001; T_2_ → T_3_: β = 0.30, *P* < 0.001; frailty: T_1_ → T_2_: β = 0.18, *P* < 0.001; T_2_ → T_3_: β = 0.26, *P* < 0.001). Loneliness at the same point in time was significantly correlated with frailty, but the correlation effect decreased over time (T_1_: β = 0.29, *P* < 0.001; T_2_: β = 0.14,* P* < 0.001; T_3_: β = 0.21, *P* < 0.001).

**Table 2:** Standardized estimates of the cross-lagged relationship between loneliness and frailty

Path	Standardized coefficient
	β(SE)
Correlation	
T_1_ loneliness $\leftrightarrow$ T_1_ frailty	0.29(0.019)^***^
T_2_ loneliness $\leftrightarrow$ T_2_ frailty	0.14(0.022)^***^
T_3_ loneliness $\leftrightarrow$ T_3_ frailty	0.21(0.022)^***^
Autoregressive	
T_1_ loneliness → T_2_ loneliness	0.25(0.026)^***^
T_2_ loneliness → T_3_ loneliness	0.30(0.024)^***^
T_1_ frailty → T_2_ frailty	0.18(0.023)^***^
T_2_ frailty → T_3_ frailty	0.26(0.022)^***^
Cross-lagged	
T_1_ loneliness → T_2_ frailty	0.03(0.016)^*^
T_2_ loneliness → T_3_ frailty	0.03(0.014)^*^
T_1_ frailty → T_2_ loneliness	0.06(0.017)^***^
T_2_ frailty → T_3_ loneliness	0.05(0.014)^***^

Note, ^***^ *P* < 0.001, ^**^ *P* < 0.01, ^*^ *P* < 0.05

Model test of the relations between change in loneliness and change in frailty

In the cross-lagged paths, the first hypothesis was supported by the positive reciprocal association between loneliness and frailty across time. T_1_ loneliness predicts T_2_ frailty significantly (β = 0.03, *P* < 0.05) and T_2_ loneliness predicts T_3_ frailty significantly (β = 0.03, *P* < 0.05), and vice versa (T_1_ frailty → T_2_ loneliness: β = 0.06, *P* < 0.001; T_2_ frailty → T_3_ loneliness: β = 0.05, *P* < 0.001). In other words, higher loneliness levels at a specific point in time significantly predict higher frailty levels in the future, and vice versa.

Table 3 shows the results of the cross-lagged model standardized estimates of the association between changes in loneliness and changes in frailty (full model results are shown in Additional Table 6). After adjusting for covariates, the model results showed that there was a significant positive correlation between loneliness and frailty, as well as changes in loneliness and frailty, at the same point in time (T_1_: β = 0.29, *P* < 0.001; Early change: β = 0.14, *P* < 0.001; Late change: β = 0.20, *P *< 0.001). Thus, at the same point in time, older adults with high levels of loneliness also tended to have higher levels of frailty, and those with deepening in frailty also tended to experience deepening loneliness.

**Table 3:** Standardized estimates of the cross-lagged relationship between change in loneliness and change in frailty

Path	Standardized coefficient
	β(SE)
Correlation	
T_1_ loneliness $\leftrightarrow$ T_1_ frailty	0.29(0.019)^***^
∆_Early_ loneliness $\leftrightarrow$ ∆_Early_ frailty	0.14(0.022)^***^
∆_Late_ loneliness $\leftrightarrow$ ∆_Late_ frailty	0.20(0.023)^***^
Autoregressive	
T_1_ loneliness → ∆_Early_ loneliness	–0.70(0.016)^***^
T_1_ loneliness → ∆_Late_ loneliness	–0.47(0.027)^***^
∆_Early_ loneliness → ∆_Late_ loneliness	–0.71(0.023)^***^
T_1_ frailty → ∆_Early_ frailty	–0.71(0.014)^***^
T_1_ frailty → ∆_Late_ frailty	–0.55(0.024)^***^
∆_Early_ frailty → ∆_Late_ frailty	–0.78(0.020)^***^
Cross-lagged	
T_1_ loneliness → ∆_Early_ frailty	0.04(0.023)^+^
T_1_ loneliness → ∆_Late_ frailty	0.04(0.025)
∆_Early_loneliness → ∆_Late_ frailty	–0.01(0.023)
T_1_ frailty → ∆_Early_ loneliness	0.03(0.018)^+^
T_1_ frailty → ∆_Late_ loneliness	0.09(0.027)^**^
∆_Early_ frailty → ∆_Late_ loneliness	0.06(0.024)^*^

*Note,* ^***^ *P* < 0.001, ^**^ *P* < 0.01, ^*^ *P* < 0.05; ^+^ *P* < 0.1

The model fit indices were CFI = 0.998, RMSEA = 0.019, and SRMR = 0.004. Combined with the previous cross-lagged model results (Table 2), the positive cross-lagged path between loneliness and frailty across time, as well as both positive autoregressive path in loneliness and frailty, suggesting that an increase in the level of loneliness or frailty at a specific time point leads to an increase in the variable itself or another variable at a subsequent time point. Therefore, we interpreted the "change" as a deepening of the level of the variable

Regarding the autoregressive path, the model results provide additional information on the trajectory of loneliness and frailty. First, T_1_ loneliness had a significant negative effect on changes in loneliness (∆_Early_ loneliness: β = –0.70, *P* < 0.001; ∆_Late_ loneliness: β = –0.47, *P* < 0.001), and early changes in loneliness also had a significant negative effect on later changes (β = –0.71, *P* < 0.001). This indicates that previous levels of loneliness had a limiting effect on subsequent changes in loneliness. Second, there were three results in the trajectory of frailty: (1) among the covariates, age had positive effects on early change and late change in the frailty (∆_Early_ frailty: β = 0.14, *P* < 0.001; ∆_Late_ ∆frailty: β = 0.13, *P *< 0.001) (details on Additional Table 6). This means that frailty in older adults may accelerate with age; (2) there were significant negative predictive relationships between T_1_ frailty and the changes in frailty (∆_Early_ frailty: β = –0.71, *P* < 0.001; ∆_Late_ frailty: β = –0.55, *P* < 0.001), as well as early changes in frailty and late changes in frailty (β = –0.78, *P* < 0.001). In other words, the older adults with high levels of frailty at the baseline have a slower rate of health loss over time; (3) the effective coefficient of T_1_ frailty for early change in frailty was –0.71, and the effective coefficient of early change in frailty for late change in frailty was −0.78, which implies that the deterioration of frailty given prior frailty in older adults is delayed. These suggests the trajectory of frailty with old age may change.

The cross-lagged relationship between T_1_ frailty and the early change in loneliness in older adults was significant at the 10% level (β = 0.03, *P* < 0.1). The late change in loneliness was significantly predicted by T_1_ frailty (β = 0.09, *P *< 0.01) and early change in frailty (β = 0.06, *P* < 0.05). This confirmed Hypothesis 2b about the effect of frailty on changes in loneliness and suggests that changes in frailty in older adults can predict subsequent changes in loneliness. In other words, older adults with high and deteriorating frailty have a faster deepening rate in loneliness at a subsequent time point.

Hypothesis 2a was not fully supported in another cross-lagged path. T_1_ loneliness positively predicted early change in frailty at the 10% significance level (β = 0.04, *P* < 0.1), while loneliness was not significantly related to late change in frailty. However, combining the significant effect of T_1_ loneliness on early change in frailty and early change in frailty on late change in loneliness produced a potentially vicious cycle of T_1_ loneliness → Early change in frailty → Late change in loneliness. This suggests that high baseline levels of loneliness in older adults may exacerbate loneliness by influencing the onset or deterioration of early frailty in older adults.

## Discussion

The present study aimed to identify the relationship between loneliness and frailty in older adults. It extended previous research by revealing the bidirectional association between loneliness and frailty among older Chinese adults and examining the relationship between changes in loneliness and changes in frailty.

In this study, we tested two hypotheses. Our first hypothesis regarding the reciprocal relationship between loneliness and frailty was fully supported. This finding is consistent with the previous studies that examined the associations separately in a single direction (e.g., [16, 27]). In addition, we found that the effect of prior frailty on subsequent loneliness was larger than the effect of prior loneliness on subsequent frailty in older adults. This may indicate that physical health has a greater influence on mental health than the converse, which is consistent with the findings of a previous study [46]. However, this finding may also have been influenced by the study period. Previous studies have already found that the effect of loneliness on the deterioration of frailty in a six-year long cohorts is greater than that in a three-year short cohorts, which suggests that the length of the survey period may affect the extent of the influence of loneliness on frailty in the elderly [17]. The length of this study was comparatively short, but the full effect of loneliness may be revealed if the study had a longer duration. However, this may also imply that there is a window period for the effect of mental health on physical health in older adults. Thus, prompting screening and assessment of mental health conditions is essential for the overall health of older adults, and more research is needed in this area.

Our second hypothesis concerns the association between changes in loneliness and changes in frailty. For Hypothesis H2a, we found an effect of baseline loneliness on the early change in frailty (*p* < 0.1), but we did not find that changes in loneliness would have significant effects on subsequent changes in frailty in older adults. This is inconsistent with previous studies that have shown a mutually influential relationship between changes in physical health and mental health [46, 47]. This may be due to the relatively short duration of this study since most of the data used in existing studies were collected over 10–21 years. However, the different findings from studies of different durations may again suggest that from a life course perspective, changes in physical health has a more pronounced effect on mental health in older adults, while the negative effects of mental health on physical health take time to manifest. Thus, early prevention of frailty may have a more influential protective function on the physical and mental health of older adults; meanwhile, the mental health assessment of older adults requires attention on an ongoing basis.

Hypothesis H2b assumed that changes in frailty was related to changes in loneliness. This hypothesis was supported by both baseline frailty and early change in frailty had significant predictive effects on late change in loneliness. Combined with the aforementioned result in Hypothesis 2a that baseline loneliness had a predictive effect on the early change in frailty was significant at the 10% level, we found that there was a potentially self-reinforcing cycle of loneliness that began with the initial level of loneliness in older adults, which then influenced the change in frailty and continued to deepen their level of loneliness. This is in line with the loneliness model proposed by John T. Cacioppo and Louise C. Hawkley [48]. The loneliness model posits that feelings of loneliness stimulate implicit hypervigilance of social threat in the environment, which triggers negative behaviors in the individual and motivates the lonely individual to actively distance himself from possible social relationships. This self-reinforcing loneliness cycle activates changes in the neurobiological and behavioral mechanisms of lonely individuals, which adversely affects their health [48, 49]. This highlights that older adults with high levels of loneliness may also be at physical health risk and may be an important mechanism by which loneliness affects the physical health of older adults. Further research may explore the underlying mechanism in the loneliness-frailty link.

Additionally, we found that age had a significant positive effect on the changes of frailty, while the prior frailty indicated a sightly slowdown exacerbation of frailty given prior frailty. These two findings may suggest that in older adults, the trend of accelerated frailty with age may change or stagnate after reaching a certain age. There are similarities between this finding and that of another study on the trajectory of frailty in older adults, which found that frailty trajectory follows a U-shaped curve and the accelerated increase in frailty with age disappeared in the eldest population (≥ 95 years old) [50]. One possible explanation is that older adults with high initial levels of frailty may be more aware of health behaviors to bridge the gap than those with low initial levels of frailty. This also implies that conscious health interventions and services can help older adults delay declines in physical function. There are inconsistent findings regarding the trajectory of loneliness in older adults [51, 52]. This study supported that high baseline loneliness had a limiting effect on the rate of deepening loneliness at subsequent times. This may have two possible explanations: (1) the change in loneliness had a baseline effect. Older adults with lower initial levels of loneliness have more room for worsening loneliness and show a higher deepening rate in loneliness over time. (2) the measurement of loneliness played a role. The limited values of loneliness variables obtained from a single question used in this study caused a ceiling effect, which limited the range of variation in loneliness.

This study also has several limitations. First, loneliness was measured by a single question in the CHARLS, which consequently limited us to examining loneliness from a single dimension. Since the prevalence of loneliness via indirect scales tends to be higher than that captured via a single question [53], we may underestimate the influence of loneliness. Second, most of the measures in this study were self-reported. Third, additional psychological factors, such as depression, were not controlled in this study. Fourth, the survey period of this study was relatively short. This may have been responsible for the weak cross-lagged effects of a reciprocal relationship between loneliness and frailty, as well as the fact that we did not find a *P* < 0.05 predictive effect of loneliness and frailty changes in older adults. Future research may use longer-term survey data to provide a more comprehensive examination of the bidirectional relationship between loneliness and frailty and the dynamics of this relationship in older adults. Furthermore, one must exhibit caution when using cross-lagged models to explain causal relationships [54]. It is possible that there is another variable that help explain the relationship between loneliness and frailty, which also calls for more research to explore the topic from multiple perspectives, including social, physiological, and psychological. Finally, since this study data are ten years old, it may not be able to directly explore the relationship between loneliness and frailty in older adults during the COVID-19 pandemic. However, the findings of this study may have more important implications for both social care policy and health intervention strategy during this particular period. Studies had shown a high prevalence of COVID-19 in older and frail patients [55, 56] and found a significant increase in loneliness in older adults during the COVID-19 pandemic  [57, 58]. The findings regarding the reciprocal relationship between loneliness and frailty highlights the need for special focus on the influence of mental health on the recovery from COVID-19 in frail older adults. This may help to improve the prognostic outcome of COVID-19 in frail elderly and also worthwhile for social care policy. Meanwhile, health intervention strategy should pay more attention to lonely older adults during the COVID-19 pandemic to prevent the possible vicious circle of loneliness and the damage to their physical health.

## Conclusions

The present study is among the first few studies to examine the longitudinal relationship between loneliness and frailty among older Chinese adults and to assess the relationship from a dynamic change perspective. It provides evidence of the reciprocal relationship between loneliness and frailty; i.e., both high loneliness leads to high frailty, and high frailty leads to high loneliness. Moreover, the findings of this study demonstrate that changes in one health domain (physical or mental) in older adults may drive changes in another health domain (mental or physical) or even trigger a vicious cycle. However, on a more positive note, this study illustrates that interventions targeting one health domain in older adults can be effective in supporting change in another. Future strategies for the health and social care system should take into account the spillover effect of health inventions. More attention should be given to early intervention for frailty in older adults and continuous screening of mental health in older adults.

## Supplementary Information

**Additional file 1: Supplementary Tables 1-6.**

## Data Availability

The datasets are publicly available from the project of the China Health and Retirement Longitudinal Study (CHARLS) and can be downloaded after registration from: http://charls.pku.edu.cn/pages/data/111/zh-cn.html
