# Supplementary material for: Associations between loneliness and frailty among older adults: Evidence from the China Health and Retirement Longitudinal Study
Source: BMC Geriatr. 2022 Jul 1;22:537. doi: 10.1186/s12877-022-03044-0 (PMC9247968; doi:10.1186/s12877-022-03044-0)
Supplement: Supplementary file 1 — Additional file 1: Supplementary Tables 1-6. [file 12877_2022_3044_MOESM1_ESM.docx]

**Supplemental table 1**

operational definition and values of five criteria for frailty in older adults

| Criteria | Definition | Values | | |
| --- | --- | --- | --- | --- |
|  |  | Female | Male | |
| weakness | gait strength | BMI≤20.6kg/m^2^:≤18.0kg | BMI≤20.0kg/m^2^:≤27.0kg | |
|  |  | 20.6＜BMI≤23.1kg/m^2^:≤18.2kg | 20.0＜BMI≤22.0kg/m^2^:≤28.5kg | |
|  |  | 23.1＜BMI≤25.7kg/m^2^:≤20.0kg | 22.0＜BMI≤24.4kg/m^2^:≤30.0kg | |
|  |  | BMI>25.7kg/m^2^:≤20.0kg | BMI>24.4kg/m^2^: ≤31.5kg | |
| walking | walking speed | height≤151cm:≤0.41m/s | | height≤162cm:≤0.47m/s |
|  |  | height >151cm: ≤0.45m/s | | height >162cm:≤0.50m/s |
| exhaustion | self-reported | felt " everything I did was an effort " or " I could not get going " about 3-4 days or 5-7 days in a week | | |
| low activity | self-reported | did not do any vigorous activities or moderate activities or walking for at least 10 min continuously during a usual week | | |
| shrinking | self-reported | lost 5 or more kilograms in the last year or weight declined 5 or more kilograms between wave 2 and wave 3. | | |

BMI: body mass index

**Supplemental table 2**

means and standard deviations for covariates

|  | 2011(T_1_) | 2013(T_2_) | 2015(T_3_) |
| --- | --- | --- | --- |
| age，N(%) |  |  |  |
| ＜65 | 1066 (44.2%) | 677 (28.1%) | 247 (10.2%) |
| 65-74 | 1094 (45.4%) | 1344 (55.7%) | 1625 (67.4%) |
| ≥75 | 252 (10.4%) | 391 (16.2%) | 540 (22.4%) |
| gender，N(%) |  |  |  |
| female | 1129 (46.8%) | 1129 (46.8%) | 1129 (46.8%) |
| male | 1283 (53.2%) | 1283 (53.2%) | 1283 (53.2%) |
| residence，N(%) |  |  |  |
| rural | 1653 (68.5%) | 1653 (68.5%) | 1653 (68.5%) |
| urban | 759 (31.5%) | 759 (31.5%) | 759 (31.5%) |
| education level，N(%) |  |  |  |
| illiterate | 730 (30.3%) | 730 (30.3%) | 730 (30.3%) |
| no formal education | 557 (23.1%) | 557 (23.1%) | 557 (23.1%) |
| elementary school | 697 (28.9%) | 697 (28.9%) | 697 (28.9%) |
| middle school or above | 428 (17.7%) | 428 (17.7%) | 428 (17.7%) |
| marital status，N(%) |  |  |  |
| without spouse | 367 (15.2%) | 440 (18.2%) | 515 (21.4%) |
| with spouse | 2045 (84.8%) | 1971 (81.8%) | 1897 (78.6%) |
| frequency of contact with children，N(%) |  |  |  |
| seldom contact | 358 (24.5%) | 318 (20.5%) | 354 (22.3%) |
| monthly contact | 361 (24.7%) | 376 (24.3%) | 412 (25.9%) |
| weekly contact | 745 (50.9%) | 856 (55.2%) | 824 (51.8%) |
| income，M(SD) | 5022.5 (10934.3) | 6216.9 (11072.5) | 5666.4 (12045.5) |
| self-rated health，N(%) |  |  |  |
| good | 491 (20.4%) | 557 (23.1%) | 463 (19.2%) |
| so so | 1180 (48.9%) | 1193 (49.5%) | 1247 (51.7%) |
| bad | 740 (30.7%) | 661 (27.4%) | 702 (29.1%) |
| number of chronic diseases，M(SD) | 1.5 (1.4) | 1.5 (1.5) | 1.9 (1.6) |
| pain，N(%)^a^ |  |  |  |
| no | 1572 (65.2%) | / | 1639 (68.0%) |
| yes | 839 (34.8%) |  | 771 (32.0%) |
| smoking，N(%) |  |  |  |
| no | 1348 (62.6%) | 1459 (65.0%) | 1725 (71.6%) |
| yes | 806 (37.4%) | 785 (35.0%) | 683 (28.4%) |
| cognitive ability，M(SD) | 12.8 (5.6) | 12.8 (5.8) | 11.7 (5.8) |
| activity participation frequency，M(SD) | 1.4 (1.9) | 1.7 (2.1) | 1.6 (2.0) |

*Note,* D = number, M = Means, SD = Standard deviation; a: because the question of pain had been removed, we did not report pain at wave 2.

**Supplemental table 3**

means, standard deviations and pairwise correlations for loneliness and frailty

|  | M (SD) | 1 | 2 | 3 | 4 | 5 | 6 |
| --- | --- | --- | --- | --- | --- | --- | --- |
| 1. T_1_ frailty | 0.9(0.9) | - |  |  |  |  |  |
| 2. T_2_ frailty | 0.7(0.8) | 0.29^***^ | - |  |  |  |  |
| 3. T_3_ frailty | 0.8(0.9) | 0.29^***^ | 0.35^***^ | - |  |  |  |
| 4. T_1_ loneliness | 1.6(1.0) | 0.29^***^ | 0.17^***^ | 0.17^***^ | - |  |  |
| 5. T_2_ loneliness | 1.4(0.9) | 0.16^***^ | 0.20^***^ | 0.11^***^ | 0.32^***^ | - |  |
| 6. T_3_ loneliness | 1.6(1.0) | 0.18^***^ | 0.17^***^ | 0.27^**^ | 0.31^***^ | 0.34^***^ | - |

*Note,* M = means, ^***^ P<0.001，^**^ P<0.01，^*^ P<0.05. The paired t-tests for frailty between waves were significant, the paired t-tests for loneliness between waves were significant except T1 and T3.

**Supplemental table 4**

variations within participants over time

|  | T1 → T2(N(%)) | T2 → T3(N(%)) | T1 → T3 (N(%)) |
| --- | --- | --- | --- |
| frailty |  |  |  |
| decrease | 812 (33.7%) | 572 (23.7%) | 717 (29.7%) |
| increase | 567 (23.5%) | 732 (30.4%) | 624 (25.9%) |
| did not change | 1033 (42.9%) | 1108 (45.9%) | 1071 (44.4%) |
| loneliness |  |  |  |
| decrease | 548 (22.7%) | 334 (13.9%) | 502 (20.8%) |
| increase | 1516 (62.9%) | 488 (20.2%) | 453 (18.8%) |
| did not change | 348 (14.4%) | 1590 (65.9%) | 1457 (60.4%) |

**Supplemental table 5**

Standardized estimates of the cross-lagged relationship between loneliness and frailty (covariates): Standardized coefficient ((β(SE)))

|  | T2 loneliness | T3 loneliness | T2 frailty | T3 frailty |
| --- | --- | --- | --- | --- |
| age | -0.05^*^  (0.021) | 0.00  (0.021) | 0.17^***^  (0.022) | 0.16^***^  (0.023) |
| gender | 0.01  (0.021) | 0.00  (0.022) | 0.08^**^  (0.027) | -0.03  (0.027) |
| residence | -0.02  (0.020) | 0.01  (0.020) | 0.01  (0.020) | -0.02  (0.020) |
| education level | -0.04  (0.022) | -0.07^**^  (0.022) | -0.08^***^  (0.023) | -0.01  (0.024) |
| marital status | -0.12^***^  (0.025) | -0.04  (0.023) | -0.01  (0.020) | 0.01  (0.022) |
| income^a^ | 0.01  (0.021) | -0.05*  (0.021) | -0.05^*^  (0.020) | -0.04  (0.021) |
| frequency of contact with children | -0.03  (0.025) | -0.08^**^  (0.026) | / | / |
| self-rated health | 0.06^**^  (0.022) | 0.07^**^  (0.021) | 0.09^***^  (0.022) | 0.05^*^  (0.021) |
| Number of chronic diseases | 0.07^**^  (0.023) | 0.03  (0.024) | 0.05^*^  (0.021) | 0.05^*^  (0.022) |
| pain | / | / | 0.05^*^  (0.022) | 0.04  (0.021) |
| smoking | / | / | -0.03  (0.025) | 0.02  (0.025) |
| cognitive ability | / | / | -0.06^**^  (0.022) | -0.10^***^  (0.022) |
| activity participation frequency | -0.03  (0.018) | -0.00  (0.018) | -0.04^*^  (0.018) | -0.02  (0.019) |

*Note,* ^***^ P<0.001，^**^ P<0.01，^*^ P<0.05; a: the logarithmic value of the economic level were used in the model.

**Supplemental table 6**

Standardized estimates of the cross-lagged relationship between change in loneliness and change in frailty (covariates): Standardized coefficient ((β(SE)))

|  | Early∆loneliness | late ∆loneliness | Early ∆frailty | Early ∆frailty |
| --- | --- | --- | --- | --- |
| age | -0.04^*^  (0.017) | -0.00  (0.020) | 0.14^***^  (0.019) | 0.13^***^  (0.020) |
| gender | 0.00  (0.017) | 0.00  (0.020) | 0.06^**^  (0.022) | -0.02  (0.023) |
| residence | -0.01  (0.016) | 0.01  (0.019) | 0.01  (0.016) | -0.02  (0.018) |
| education level | -0.03  (0.018) | -0.05^**^  (0.020) | -0.07^***^  (0.019) | -0.01  (0.021) |
| marital status | -0.10^***^  (0.020) | -0.01  (0.021) | -0.01  (0.017) | 0.01  (0.020) |
| income^a^ | 0.01  (0.017) | -0.04^*^  (0.019) | -0.04^*^  (0.017) | -0.03  (0.019) |
| frequency of contact with children | -0.03  (0.020) | -0.05^*^  (0.024) | / | / |
| self-rated health | 0.05^**^  (0.018) | 0.03  (0.020) | 0.07^***^  (0.018) | 0.02  (0.019) |
| Number of chronic diseases | 0.06^**^  (0.019) | 0.02  (0.022) | 0.04^*^  (0.018) | 0.04^*^  (0.020) |
| pain | / | / | 0.04^*^  (0.018) | 0.02  (0.019) |
| smoking | / | / | -0.03  (0.021) | 0.01  (0.022) |
| cognitive ability | / | / | -0.05^**^  (0.018) | -0.07^***^  (0.019) |
| activity participation frequency | -0.03  (0.020) | 0.00  (0.017) | -0.03^*^  (0.015) | -0.01  (0.017) |

Note, ^***^ P<0.001，^**^ P<0.01，^*^ P<0.05; a: the logarithmic value of the economic level were used in the model.
